# Coupling of PZT Thin Films with Bimetallic Strip Heat Engines for Thermal Energy Harvesting

**DOI:** 10.3390/s18061859

**Published:** 2018-06-06

**Authors:** Jihane Boughaleb, Arthur Arnaud, Benoit Guiffard, Daniel Guyomar, Raynald Seveno, Stéphane Monfray, Thomas Skotnicki, Pierre-Jean Cottinet

**Affiliations:** 1 STMicroelectronics (Crolles 2) SAS, 850 Rue Jean Monnet, 38926 Crolles CEDEX, France; arthur.arnaud-ext@st.com (A.A.); stephane.monfray@st.com (S.M.); thomas.skotnicki@st.com (T.S.); 2 Univ Lyon, INSA-Lyon, LGEF, EA682, F-69621 Villeurbanne, France; daniel.guyomar@insa-lyon.fr (D.G.); pierre-jean.cottinet@insa-lyon.fr (P.-J.C.); 3 CEA Liten, 17 Rue des Martyrs, 38054 Grenoble CEDEX 9, France; 4 Université Bretagne Loire, Université de Nantes, IETR UMR CNRS 6164, 44322 Nantes, France; benoit.guiffard@univ-nantes.fr (B.G.); raynald.seveno@univ-nantes.fr (R.S.)

**Keywords:** energy harvesting, bimetallic strip, heat engine, PZT thin films

## Abstract

A thermal energy harvester based on a double transduction mechanism and which converts thermal energy into electrical energy by means of piezoelectric membranes and bimetals, has previously been developed and widely presented in the literature In such a device, the thermo-mechanical conversion is ensured by a bimetal whereas the electro-mechanical conversion is generated by a piezoelectric ceramic. However, it has been shown that only 19% of the mechanical energy delivered by the bimetal during its snap is converted into electrical energy. To extract more energy from the bimetallic strip and to increase the transduction efficiency, a new way to couple piezoelectric materials with bimetals has thus been explored through direct deposition of piezoelectric layers on bimetals. This paper consequently presents an alternative way to harvest heat, based on piezoelectric bimetallic strip heat engines and presents a proof of concept of such a system. In this light, different PZT (Lead zirconate titanate) thin films were synthesized directly on aluminium foils and were attached to the bimetals using conductive epoxy. The fabrication process of each sample is presented herein as well as the experimental tests carried out on the devices. Throughout this study, different thicknesses of the piezoelectric layers and substrates were tested to determine the most powerful configuration. Finally, the study also gives some guidelines for future improvements of piezoelectric bimetals.

## 1. Introduction

**Figure 1:**
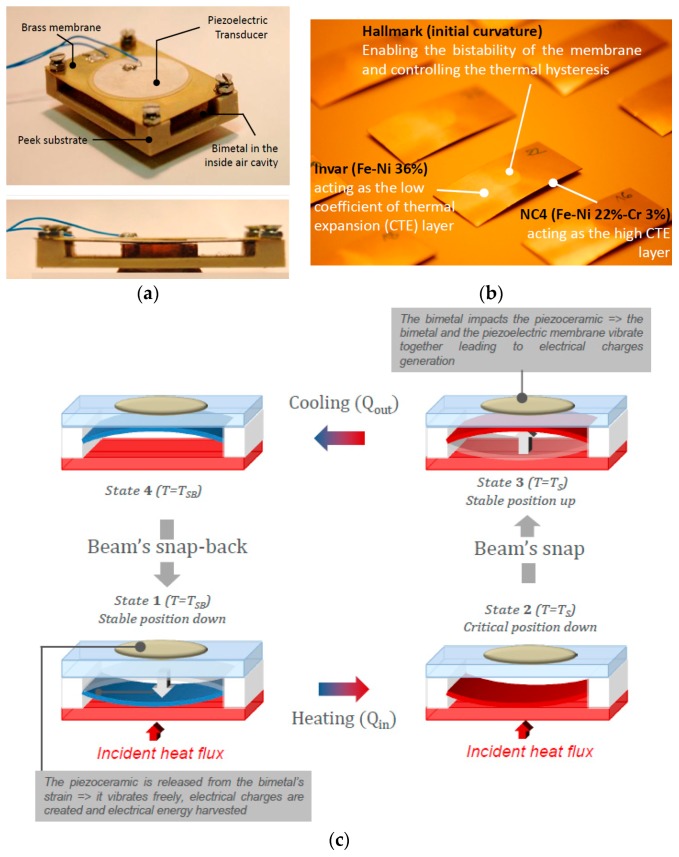
(**a**) Thermal energy harvester previously developed based on a piezoelectric membrane and a bimetallic strip in the inside air cavity; (**b**) Image of the precurved bimetal; (**c**) Working principle of the harvester and different states of the bimetal.

Energy harvesting using piezoelectric [1,2,3,4,5,6], thermal [7,8,9] or electrostatic [10] conversion is pursued as an alternative to batteries for the power supply of wireless sensor nodes. Nowadays, emerging sensor nodes have lower and lower consumptions and emerging energy harvesting systems are able to provide enough energy to power them. The typical consumption of an autonomous smart sensor is comprised between 1 µW and 20 µm thanks to the reduction in size and consumption of CMOS electronics. In many domains, such as for instance the industrial or health sectors, attention has been brought to the abundant thermal energy that can be harvested. In this context, the thermal energy harvester shown in Figure 1 has been developed. This device is based on a two-step conversion mechanism: a thermo-mechanical conversion by a bimetallic strip followed by an electro-mechanical conversion thanks to a piezoelectric membrane placed on the bimetal. This configuration, first proposed by Skotnicki in [11], has been widely studied in the literature [1,2,3,4]. 

As shown in Figure 1a,c, the bimetallic strip is mounted between two heat sources of different temperatures. When it comes in contact with the hot source (State 1 Figure 1c), the bimetal is at its first stable position. It is heated and a bending moment is generated. Once a certain value is reached, the bimetal snaps up to a second stable position where it comes into contact with a cold surface (from state 2 to 3 in Figure 1c). At this point in time, the bimetal is cooled down and a bending moment in the opposite direction is again generated (from state 3 to 4 in Figure 1c). Once a second threshold value is achieved, the beam snaps down (from state 4 to 1 in Figure 1c). This way, the bimetal behaves like a self-oscillating mechanical system cycled between two surfaces as shown in Figure 1c. 

This whole cycling concerns the thermo-mechanical conversion part of the structure and creates an oscillating thermal field inside the harvester. To generate electrical charges from the bimetal’s thermo-mechanical snapping, a piezoelectric membrane is placed above the bimetal and is also used as a cold surface. Each bimetal snap transmits a pulse of energy to the piezoelectric that converts it into electric energy. However, it should be noticed that each bimetal has its own thermal hysteresis: a bimetal snaps up when its active part (hallmark in Figure 1b) reaches the snap temperature T_S_ and snaps back when its temperature reaches the snap-back temperature T_SB_. The difference between these two temperatures gives the bimetal’s hysteresis.

In [4], the author established a dynamic thermal model of the device taking into account the oscillating bimetal inside the system and all the heat exchanges occurring inside it. This model makes it possible to carry out calculations on the dynamic behaviour of each bimetal, with a certain snapping temperature and hysteresis and as a function of the ambient air temperature and the hot source temperature acting as the input of the experiment for a permanent bimetal thermal cycling.

To understand how it is possible to harvest energy thanks to bimetals, it is necessary to explain the origins of their thermo-mechanical bi-stability. Bimetal membranes are basically made of two materials with a mismatch in their thermal expansion coefficients (TECs) (Figure 1b). This makes them bend when subjected to temperature variations. Initially, this asymmetry of the membrane’s thermal properties is not sufficient to produce a bi-stable behaviour, but, as explained by Wittrick in the case of bimetallic shells [12], by combining the membrane with another kind of asymmetry involving an antagonistic effect such as an initial curvature, a mechanical instability starts to appear, characterized by the existence of a thermal hysteresis. 

**Figure 2:**
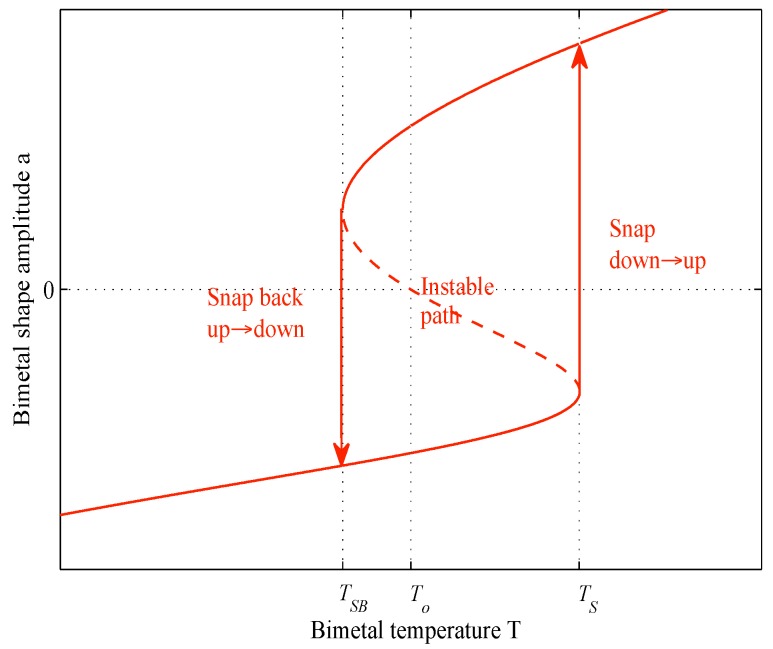
Hysteretic behaviour of bimetals.

As illustrated in Figure 2, when the bimetal temperature rises and reaches the snap temperature T_S_, the bimetal switches from an unstable down-state to a stable up-state, releasing part of its thermal strain energy as kinetic energy. Once the bimetal has snapped, if its temperature decreases and reaches the snap-back temperature T_SB_ (with T_SB_ < T_S_), the membrane switches back to its initial state, again releasing kinetic energy. 

After several optimization steps of the coupled piezoelectric and bimetallic strip heat engines, as shown in Figure 1 (including thermal optimization of the thermal gradient across the hot source and the cold surface, device architecture optimization from both mechanical and thermal points of views and also pertinent piezoelectric material choices), the output power was increased from 1 µW [1] (generated by the first prototype developed for the proof of concept) to 30 µW available on the piezoelectric capacitor for a hot source of 84 °C and a 3 °C hysteretic bimetal (67 °C/70 °C) [12]. This corresponds to 5 µW of usable power when employing a standard electronic circuit composed of a diode bridge and a storage capacitor. Since the purpose of developing such a harvester was to supply an autonomous wireless sensor node (WSN), tests were carried out using a GreenNet WSN and an output power of 4 µW was found to be sufficient for emitting data from the emission module toward the reception once every 30 s with a consumption of 120 µW per emission [12].

This paper explores a new means of coupling bimetals with piezoelectric materials through direct deposition of PZT thin films on bimetals. The main advantage of this technique is to be able to continuously harvest energy: heat will be harvested during both the bimetal’s snapping and snapping back but also during its cooling and heating.

## 2. Fabrication and Testing of Piezoelectric Bimetals

### 2.1. Fabrication Procedure of Thin PZT Films

Several materials such as Pb(Zr,Ti)O_3_ (PZT), ZnO or AlN can be used for the fabrication of piezoelectric thin films. Due to the superior piezoelectric properties of PZT, this material was the one used here and highly flexible lead zirconate titanate thin films were realized by a sol-gel process. The PZT thin films were first synthetized on aluminium foils and were finally attached on the bimetals. In fact, commercial aluminium foils present numerous advantages, including an ultralight weight (43 g·m^−2^), flexibility and conformability, conduction, low pricing (less than 0.1 $·m^−2^) and a low Young modulus (69 GPa).

**Figure 3:**
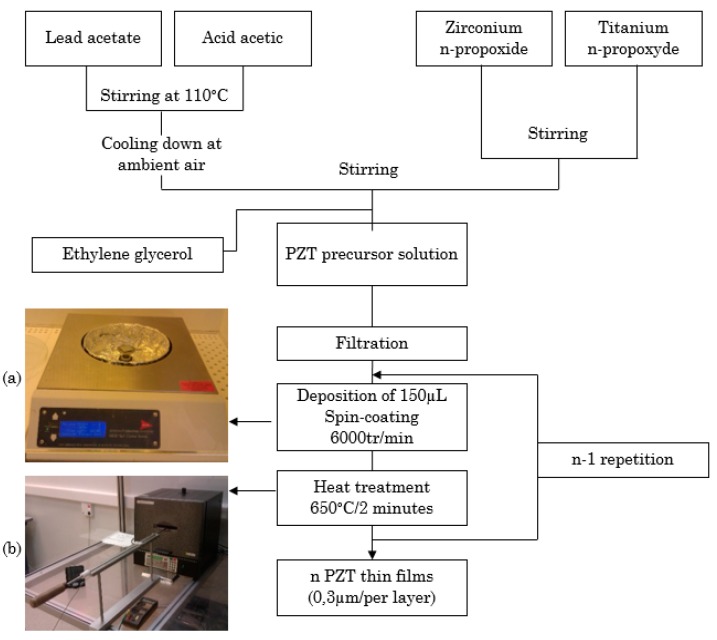
Deposition process of the PZT thin films (**a**) Spin coating equipment; (**b**) Oven used for the heat treatment of the thin films.

For the fabrication of PZT thin films the same procedure as the one presented in [13,14,15,16] was followed and Figure 3 presents the deposition process on Al foils. For bulk PZT materials, the maximum ferroelectric and piezoelectric properties were obtained at the morphotropic (MPB) phase boundary composition [17]. This composition was found for a Zr/Ti ratio close to 53/47 [18]. Consequently, the Zr/Ti ratio used for the solution preparation was of 57/43. Different materials were employed for the fabrication of the thin films including:

- Lead acetate (Pb(CH_3_CO_2_)_2_·3H_2_O, Alfa Aesar), zirconium n-propoxide (Zr(C_3_H_7_O)_4_, Alfa Aesar, 70%) and titanium n-propoxide (Ti(C_3_H_7_O)_4_, Adrich, 98%) used as precursor materials.
- Acetic acid used as a solvent.
- Ethylene glycol (C_2_H_6_O_2_, Fluka, 99.5%) used to prevent cracks during the crystallization of the thin films.

Lead acetate was first dissolved in heated acetic acid. Zirconium and titanium were mixed together and then added to the lead acetate solution. Ethylene glycol was finally incorporated into the solution. The precursor solution was deposited onto aluminium foils with different thicknesses by spin coating at 6000 tr/min during 20 s to obtain a PZT film thickness of 300 nm. The coated layer was then introduced in a furnace at 650 °C during 2 min to improve the crystallinity of the PZT films while keeping the aluminium foil safe. Depending on the targeted thickness of the PZT thin films, the same procedure was repeated for each 300 nm layer. The aluminium foils used for this experiment had thicknesses of 15 µm or 30 µm while the deposited PZT thin films were 2, 3 or 4 µm thick. For the electric measurements and poling, a 150 nm thick aluminium top electrode with an area of 10 mm × 3 mm was sputtered. Once the PZT thin films were ready, they were glued to the bimetals using electrically conductive epoxy.

**Figure 4:**
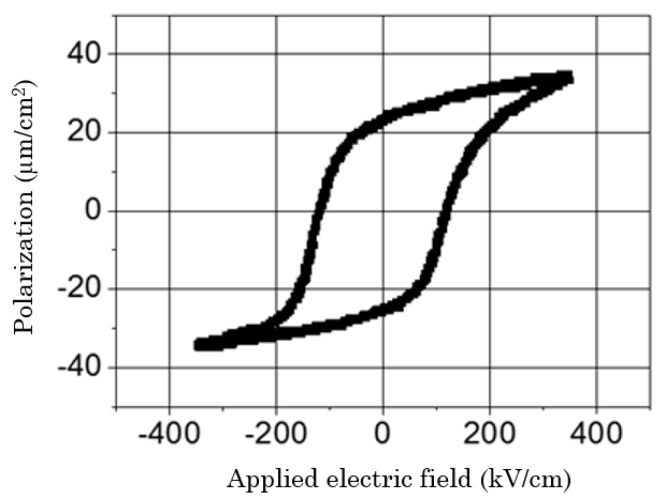
Hysteresis loop (P(E)) of a 4 µm PZT film deposited onto a 30 µm aluminium foil.

To exhibit macroscopic piezoelectric properties, the samples were poled. A Sawyer-Tower circuit was employed for the poling and to investigate the ferroelectric hysteresis cycle for each device. An electric field of 360 kV/cm at 50 Hz was applied to the samples. Figure 4 shows the hysteresis loop of a PZT thin film of 4 µm deposited onto an aluminium foil of 30 µm.

### 2.2. Properties of Piezoelectric Bimetals

The goal of this study was to investigate the electrical power delivered by a piezoelectric bimetal using PZT thin films. To do so, the impact of the aluminium and the PZT film thicknesses on the output power was explored as was the number of PZT films deposited per bimetal. Two kinds of bimetals were used in the experiment: 8 bimetals of 6 K hysteresis and 9 bimetals of 12 K hysteresis. All the bimetals had the same snapping temperature of 70 °C and the same dimensions: 0.3 mm thick, 36 mm long and are 18 mm wide. Moreover, their weight was about 0.6104 g. Some bimetals had only one PZT thin film on it while others had 2 PZT layers for the purpose of comparison. In this last configuration, the two layers were poled in an antiparallel configuration and were connected in a series configuration to reduce the equivalent capacitance and increase the output piezoelectric voltage. Table 1 sums up all the devices realized and their characteristics in terms of the number of PZT thin film layers and their thicknesses, the aluminium substrate thicknesses and finally the bimetals’ hysteresis.

**Figure 5:**
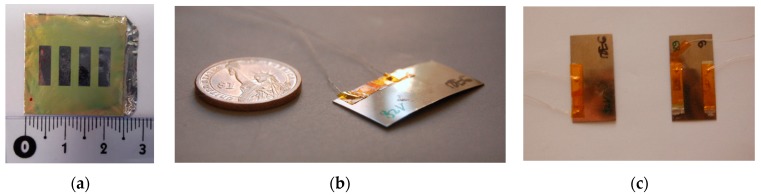
(**a**) Top view of the realized PZT thin films; (**b**) Piezoelectric bimetal with one PZT thin film; (**c**) Piezoelectric bimetals with one and two PZT thin films.

Figure 5a shows a top view of the fabricated PZT thin films on an aluminium foil with 4 deposited electrodes. Figure 5b shows a bimetal with one Al/PZT thin film and Figure 5c shows bimetals with one and two Al/PZT thin films. 

### 2.3. Characterization of the Piezoelectric Bimetals

**Figure 6:**
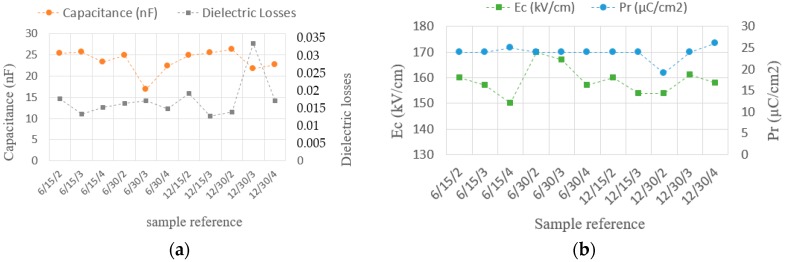
(**a**) Values of the capacitance and the dielectric losses of the fabricated samples; (**b**) Coercive field and remnant polarization of the fabricated samples.

The capacitance value of each thin film, its dielectric losses, its remnant polarization and its coercive field were measured and shown in Figure 6a,b for the bimetals having only one PZT layer. In the x-axis of these graphs, the reference of each sample is given. The format of the reference is: bimetal hysteresis (K)/Al foil thickness (µm)/PZT film thickness (µm). For example, the reference 6/15/2 refers to the 6 K hysteresis bimetal, with a deposited PZT thin film fabricated using a 15 µm aluminium foil and a PZT film thickness of 2 µm.

**Figure 7:**
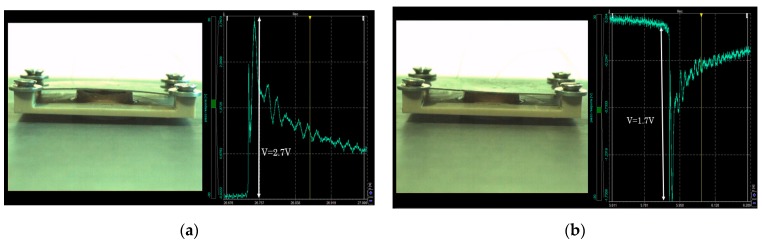
(**a**) Electrical signal of the piezoelectric bimetal during its snap-up (sample 6/30/4) and (**b**) during its snap back.

The fabricated PZT layers had a mean capacitance of 25 nF. To observe the open circuit voltage of each piezoelectric bimetal during its heating and cooling phases, a voltage follower circuit (TL082IP, Texas Instrument, Dallas, TX, USA) whose input impedance was equal to 10^12^ ohms was employed. Each bimetal was set in a peek substrate like those previously used in order to maintain the bimetal in its cavity (same experimental setup as in Figure 1). Figure 7a presents the signal delivered by the piezoelectric bimetal snap signal and Figure 7b the snap-back signal. These images were realized using a high resolution and synchronized camera with the following reference: DS-CAM-600C (DEWE, Paris, France) from DEWESOFT (DEWE, Paris, France). 

To fix the functioning temperature, the dynamic thermal model in [4] was used. Given that the ambient air temperature for this experiment was 25 °C and that all the bimetals had the same snapping temperature of 70 °C, the hot source temperature was set at 84 °C.

Subsequently, the impact of both the PZT and aluminium films thicknesses on the output power was studied. In both cases, the investigation involved one and two PZT layers per bimetal. Then, the devices with one and two layers were compared and the most powerful configuration was pointed out. 

## 3. Results and Discussions

### 3.1. Performance Comparison of the Bimetals with a Single PZT Thin Film

**Figure 8:**
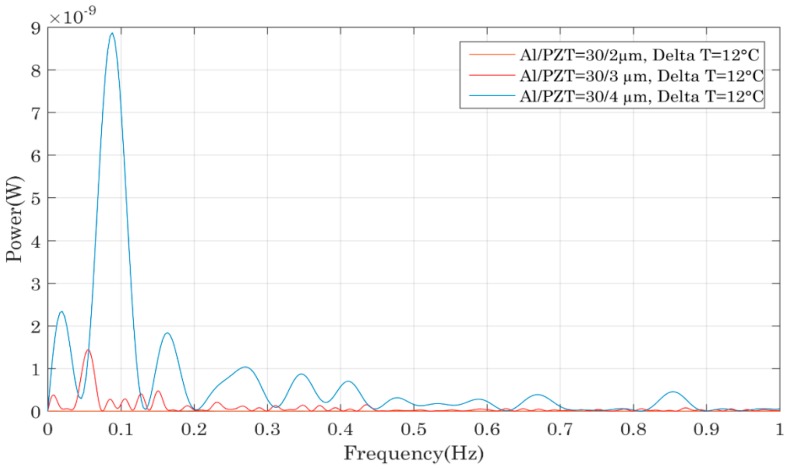
Impact of the PZT thin film thickness on the output power of the piezoelectric bimetal mounted in the device as shown in Figure 7.

**Figure 9:**
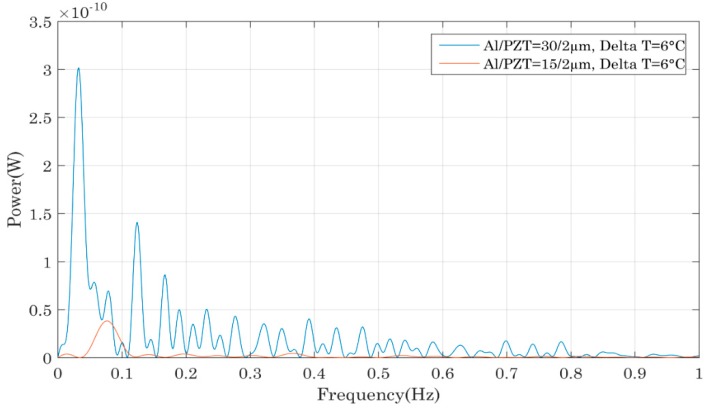
Impact of the Al foil thickness on the output power of the piezoelectric bimetal mounted in the device shown in Figure 7.

**Figure 10:**
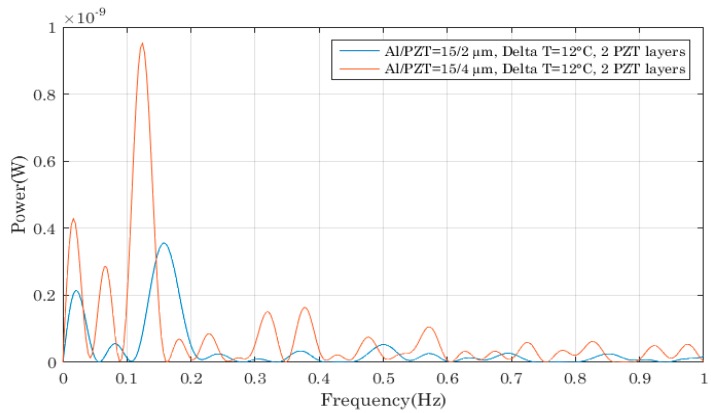
Impact of the PZT film thicknesses on the output power of the piezoelectric bimetal with two PZT thin films.

**Figure 11:**
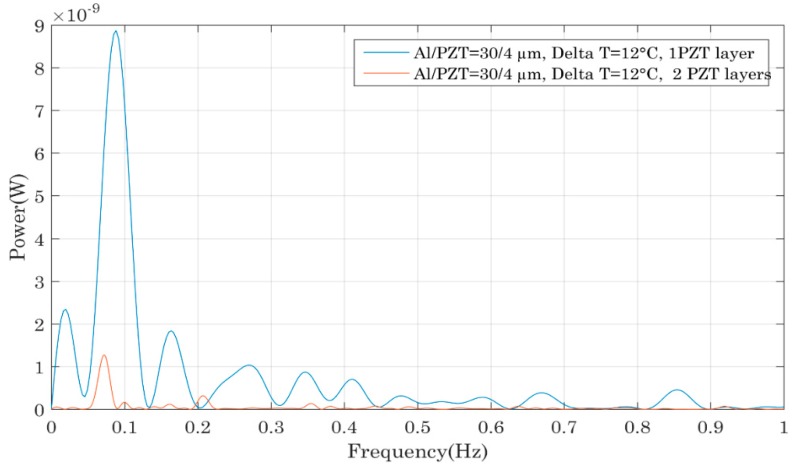
Impact on the device’s output power of the number of PZT thin films deposited onto the bimetal.

The calculation of the power output in an open circuit configuration for each device was realized using the signal’s FFT (Figure 8, Figure 9, Figure 10 and Figure 11). To access the instantaneous output power across an adapted charge, the same technique as in [19,20,21,22] is used. The FFT measurements enabled us to determine the frequential spectrum of the output voltage and power and especially at which frequency it was possible to extract the maximum power with a load resistance. Knowing this frequency, we have been able to deduct the optimal resistance maximizing the extraction of the energy of the piezoelectric capacitor. To experimentally find that optimal load resistor, different resistances are tested and the optimum power transfer is achieved for R_opt_ = 1/C_piezo_·ω_res_ where C_piezo_ the PZT thin film capacitance and ω_res_ its resonance frequency. Using this adapted resistance that matches the impedance of the piezoelectric capacitor, we find that the output voltage is equally divided between the capacitor and the load resistance. In this case, the energy dissipated in the optimal load resistor is proportional to ¼ times the square of the effective voltage, so 1/8 times the square of the maximum voltage across the capacitor. Consequently, thanks to the comparison of the output power in open circuit configuration, one is able to access to the useful power across an adapted load. In each case, the compared bimetals had the same hysteresis and only the thickness of the Al or PZT films varied. In Figure 8, the bimetals had a hysteresis of 12 °C, the Al foil was 30 µm tick and the PZT thickness varied from 2 to 4 µm. Two other groups of bimetals were studied: three bimetals whose hysteresis equalled 6 °C, with an aluminium thickness of 30 µm and different PZT thicknesses of 2, 3 and 4 µm, in addition to two bimetals with a 6 °C hysteresis, aluminium foil of 15 µm and PZT thicknesses of 2 or 4 µm. For all the compared devices, we observed the same evolution: the power increased with the PZT thickness, which was logical since a thicker PZT layer led to the generation of more electrical charges and thus a higher amount of electrical power. The generated power was comprised between 0.1 nW for the sample 6/15/2 and up to 10 nW for the sample 6/30/4.

Figure 9 compares the output power of different piezoelectric bimetals as a function of the aluminium foil thickness. Like in the previous comparison, the bimetals had the same thermal hysteresis and PZT film thickness but the aluminium foil thickness varied. The samples compared in this graph had the following references: 6/15/2 and 6/30/2. In addition to this group of samples, we also compared the bimetals 6/15/4 and 6/30/4, followed by the bimetals 12/15/3 and 12/30/3. The three comparisons showed that a thicker aluminium foil allowed more power generation. Such a trend was due to the position of the neutral axis across the beam: the stress distribution across the bimetal’s thickness made it more interesting to have a thicker aluminium foil as the PZT layers becomes located in a region of higher stress distribution which led to higher electrical power. Consequently, the generated power reached a maximum of 3 nW for the sample 6/30/2 and less than 0.1 nW for the sample 12/15/2.

From this first comparison established for one PZT layer per bimetal, it seemed that a thick PZT layer and a thick aluminium foil made it possible to harvest more power, since the best configuration was the one where the PZT layer was 4 µm thick with an aluminium thickness equal to 30 µm.

### 3.2. Performance Comparisons of the Bimetals with Two PZT Thin Films

Next, we explored whether the previous observations could be verified if two PZT layers were used on each bimetal. Figure 10 compares different device performances as a function of the PZT thickness and we came to the same conclusion as previously: a higher PZT layer allowed the generation of more charges and thus more power scavenging.

We then compared different bimetals, varying only the aluminium thickness. Contrary to the comparison established previously when one PZT layer was deposited on each bimetal, no trend could be observed when two thin films were deposited per bimetal. This was related to the bimetals’ thermal properties changing when thick additional layers were deposited on them. In fact, when many layers were placed on the bimetal, its global thickness increased affecting the ratio between the bimetal’s thickness and its curvature. In [23,24], the author gives criterions for bimetals’ bi-stability, corresponding to a certain ratio between thickness and curvature and demonstrates that the bimetal’s hysteresis is lowered when its thickness increases, this leading to less mechanical energy per snap. Moreover, if the bimetal’s thickness keeps on increasing, this could even lead to the system losing its bi-stability. 

The reason we do not observe such behaviour in Figure 10 is because the thickness of the PZT film was only varied from 2 to 4 µm, whereas for the aluminium foil, the thickness went from 15 to 30 µm which is at least 7 times higher. 

### 3.3. Performance Comparisons of Bimetals with One and Two PZT Thin Films

This last part presents a comparison of the performances of bimetals having the same properties and identical PZT layers except for one bimetal that had only a single PZT layer whereas the other had two. The graph of Figure 11 shows that the bimetals with only one PZT layer were more powerful and the same observation was made for the samples 6/30/3 with one and two PZT films. This seemed to be counter-intuitive because more PZT was deposited on each bimetal so more power was expected to be generated. But the reason of such a behaviour was again related to the bimetal properties. Arnaud studied bimetal modelling during his PhD work and his findings allow us to explain the observed trend [23,24]. As previously mentioned, for bi-stable bimetals with a certain hysteresis, one of the parameters that fixes the hysteresis value is the ratio between the thicknesses of the bimetal and its curvature. When additional layers are deposited onto the bimetal, its general thickness is increased and thus its hysteresis is lowered [23]. This leads to a lower mechanical force during snapping and consequently to less electrical power.

From all the comparisons established here, the most powerful bimetal was the one with a single PZT film whose thickness equalled 4 µm on a 30 µm thick aluminium foil. This device generated an electrical power of nearly 10 nW in open circuit configuration corresponding to a maximum of 2.5 nW of useful power across an adapted charge. So, to conclude, PZT thin films render possible the harvesting of electrical energies in the range of a few nano-watts if thick Al foils and PZT layers films are used. However, one should keep in mind that only thin PZT films can be deposited on each bimetal so as not to drastically change the thermal properties: otherwise the thermal hysteresis can be lowered or the bi-stability can even be lost. For this reason, it is important not to use bimetals with small thermal hysteresis to avoid loss of their bi-stability but also to not entirely cover the surface of the bimetal with thin film. This study has allowed us to demonstrate the feasibility of this coupling. However, since the amount of harvested power did not exceed 10 nW for the most powerful system, it is important to carry out further studies with regard to film deposition optimization and harvested energy improvement before performing a wireless sensor node demonstration as in [25]. 

## 4. Conclusions

This paper describes the investigation of a new way to couple bimetals with piezoelectric materials for thermal energy harvesting. We explored direct deposition of piezoelectric materials on bi-stable bimetals thanks to massive PZT thin films. The advantages of this new coupling included the ease of processing, the low cost and the capability to harvest both the piezoelectric and the pyroelectric charges generated during heating, cooling and snapping of the bimetals. For these first demonstrations, we tested bimetals with one or two PZT thin films each and the characterization results showed that the bimetals with one piezoelectric film had superior performances. It was also demonstrated that thick PZT layers and Al layers allowed more charges to be generated. Up to now, in the best case, we were able to extract a maximum of 10 nW using a 6 K bimetal with a PZT film whose thickness equalled 4 µm on a 30 µm thick aluminium foil. These first results exhibited lower performances compared to the devices with piezoelectric membranes and also highlighted the piezoelectric bimetals’ limitation when it comes to thick deposited layers. Nevertheless, the results were encouraging for future developments as this technique remains of great interest for an easier integration and size reduction.

To improve the amount of harvested thermal power, two main axes should be investigated regarding the PZT films: the exploration of the maximum PZT thickness allowed without changing the bimetal’s hysteresis and the study of the best positioning of the PZT thin film on the bimetal to position it in an area of maximal stress distribution each time the bimetal snaps. 

Finally, the performances can also be optimized by acting on the bimetals’ intrinsic properties: the currently used bimetals were realized with materials having low thermal conductivities which led to higher heating and cooling times and consequently to low snapping frequencies as seen on the FFT curves and power outputs. To avoid this drawback and improve the harvested energy, efforts are being made by bimetal manufactures to change the materials and replace them with new ones having at the same time a significant mismatch of their coefficients of thermal expansion and good thermal conductivities to quicken the thermal heat exchanges.

## Figures and Tables

**Table 1:** Properties of the PZT thin films deposited onto the bimetals, T_S_ = 70 °C for all bimetals.

	Al/PZT	15/2 µm	15/3 µm	15/4 µm	30/2 µm	30/3 µm	30/4 µm
1 PZT layer per bimetal	Bimetal ∆T = 6 K	X	X	X	X	X	X
	Bimetal ∆T = 12 K	X	X		X	X	X
2 PZT layers per bimetal	Bimetal ∆T = 6 K		X			X	
	Bimetal ∆T = 12 K	X		X	X		X
